# Predictive models for IOPs measured with NCT, GAT, and ORA among patients undergoing SMILE

**DOI:** 10.3389/fbioe.2022.1030458

**Published:** 2022-12-01

**Authors:** Tian Han, Wanru Shi, Yingjun Chen, Yang Shen, Ye Xu, Xingtao Zhou

**Affiliations:** ^1^ Department of Ophthalmology, Eye and ENT Hospital of Fudan University, Shanghai, China; ^2^ NHC Key Laboratory of Myopia Fudan University, Shanghai, China; ^3^ Research Center of Ophthalmology and Optometry Shanghai, Shanghai, China; ^4^ Shanghai Engineering Research Center of Laser and Autostereoscopic 3D for Vision Care (20DZ2255000), Shanghai, China

**Keywords:** small incision lenticule extraction (SMILE), intraocular pressure (IOP), noncontact tonometer (NCT), Goldmann applanation tonometry (GAT), ocular response analyzer (ORA)

## Abstract

**Purpose:** To develop predictive models for the intraocular pressure (IOP) of patients undergoing small incision lenticule extraction (SMILE) procedures, measured with a noncontact tonometer (NCT), Goldmann applanation tonometry (GAT), and an ocular response analyzer (ORA).

**Methods:** In this prospective study, a total of 104 eyes (−6.23 ± 2.06 diopters) of 52 patients (24.38 ± 4.76 years) undergoing SMILE procedures were included. The intraocular pressure was measured (IOP_NCT_ with NCT, IOP_GAT_ with GAT, and IOPcc and IOPg with ORA) before surgery and at postoperative 6 months. Information on age, preoperative and attempted spherical equivalent (SE), ablation depth, preoperative values and postoperative changes in central corneal thickness (CCT), K1, K2, Km, corneal hysteresis (CH) and corneal resistance factor (CRF) values was collected in order to predict IOPs.

**Results:** All surgeries were uneventful. At postoperative 6 months, the efficacy and safety index were 1.04 ± 0.15 and 1.08 ± 0.18, respectively. Significant decreases were detected in postoperative IOP_NCT_, IOP_GAT_, IOPcc, and IOPg compared to preoperative values (all *p* < 0.001). No relationship was found between any IOP and ablation depth, attempted SE, and preoperative SE, as well as CCT_difference_ (all *p* > 0.05). Predictive models for IOPs were constructed to predict preoperative values, and *R* ^2^ values were 67.5% (IOP_NCT_), 64.5% (IOP_GAT_), 78.7% (IOPcc), and 82.0% (IOPg). The prediction band of IOP_NCT_ and IOP_GAT_ was 7.4–15.1 mmHg and 8–16 mmHg, respectively.

**Conclusion:** Predictive models for IOP measurements after SMILE procedures can be helpful in clinical practice.

## Introduction

In clinical practice, it is important to monitor the intraocular pressure (IOP), especially for patients undergoing refractive surgeries. IOP management after refractive surgeries calls for the avoidance of steroid-induced hypertension and an accurate diagnosis of glaucoma, since myopia is a risk factor for open-angle glaucoma (Marcus et al., 2011).

Factors such as central corneal thickness (CCT), corneal curvature, and corneal biomechanics affect IOP measurements (Okafor and Brandt, 2015). Both Goldmann applanation tonometry (GAT), a standard measurement for normal corneas, and noncontact tonometer (NCT), a commonly used instrument, can underestimate the IOP after refractive surgeries due to the corneal tissue being removed during surgeries (Ehlers et al., 1975; Ito et al., 2012; Schallhorn et al., 2015). The ocular response analyzer (ORA) provides Goldmann IOP (IOPg), corneal-compensated IOP (IOPcc) (Moreno-Montanes et al., 2008), and corneal biomechanics: corneal hysteresis (CH) and corneal resistance factor (CRF) (Medeiros and Weinreb, 2006). Corneal biomechanics may be helpful in predictive IOP models (Li et al., 2016).

There are many studies on IOP among patients undergoing laser-assisted *in situ* keratomileusis (LASIK) and photorefractive keratectomy (PRK) (Chang and Stulting, 2005; Kohlhaas et al., 2006; Yang et al., 2006; Pepose et al., 2007; Johannesson et al., 2012; Han et al., 2013; Schallhorn et al., 2015; Sales-Sanz et al., 2016), while limited literature reports exist on predictive models of IOP changes after small incision lenticule extraction (SMILE) (Li et al., 2016; Shen et al., 2016). As a flapless procedure utilizing a small incision, SMILE preserves the integrity of the corneal tissue (including Bowman’s layer) and serves a better corneal biomechanics than LASIK (Wang et al., 2022), although it does not preserve suspect corneas before iatrogenic ectasia (Bao et al., 2022; Xin et al., 2022). The impact of SMILE on the corneal structure prompts changes in the IOP that might differ from other refractive surgeries. In addition, IOP changes should be focused for the fact that the number of SMILE surgeries has currently reached 5 million globally.

In this prospective study, we developed predictive models for four IOPs among patients undergoing SMILE procedures.

## Materials and methods

Patients who underwent SMILE procedures at the Refractive Surgery Center of the Department of Ophthalmology, Eye and ENT Hospital of Fudan University, were enrolled in this prospective study. This study was registered in the Chinese Clinical Trial Registry (ChiCTRONRC13003114), followed the tenets of the Declaration of Helsinki, and was approved by the ethics committee of the Eye and ENT Hospital of Fudan University. Informed consent was obtained from all participants.

Inclusion criteria included an age of 18–48 years, myopia with a spherical equivalent (SE) less than 12.50 diopters (D), corrected distance visual acuity (CDVA) of 20/20 or better, a stable refraction for 2 years, IOP_GAT_ between 10 and 21 mmHg, and no use of any kind of contact lenses within the previous 2 weeks. Patients with any systemic diseases, any history of ocular surgery or trauma, or any history of ocular disease except myopia or astigmatism were excluded.

### Study procedures

Except for normal preoperative examinations, the IOP_NCT_ of all patients was measured using a noncontact tonometer (NCT) (TX-F; Canon, Tokyo, Japan), IOP_GAT_ by Goldmann applanation tonometry (GAT) (Haag-Streit, Bern, Switzerland), and IOPcc and IOPg using an ocular response analyzer (ORA) (Reichert, Inc., Depew, NY, United States) between 9 and 12 o’clock in the morning. In all cases, each IOP was measured three times by experienced clinicians.

The same surgeon (XZ) performed all SMILE procedures. The 500-kHz VisuMax femtosecond laser system (Carl Zeiss Meditec, Jena, Germany) was used with a pulse energy of 130 nJ. The lenticule diameter was set between 6.00 and 6.80 mm; the cap diameter was set to 7.5 mm at a 120 μm depth. A 90-degree single side cut with a length of 2.0 mm was created during the procedure.

The postoperative follow-up was set at 6 months. At postoperative 1 month, all patients were told to stop medication such as steroids, which can affect the IOP. Routine examinations such as uncorrected distance visual acuity (UDVA), CDVA, and manifest refraction were performed. Information on CCT, K1, K2, and Km was collected by rotating Scheimpflug camera imaging (Pentacam; Oculus GmbH, Wetzlar, Germany).

### Data analysis

All statistical analyses were performed using the Statistical Package for Social Sciences (SPSS, Version 22) (IBM, Armonk, NY, United States) and Statistical Analysis Software (SAS Version 9.4, SAS Institute, Cary, NC, United States). Data were reported as the mean ± standard deviation. A generalized linear mixed model (GLMM) was used to build predictive models of IOP_difference_ and to detect the difference between the four IOPs with the inter-eye correlation. From the model constructed using the GLMM, the goodness-of-fit statistic R2 was calculated using a least-square method. For all tests, *p* < 0.05 was defined as statistically significant.

## Results

A total of 104 eyes of 52 patients (30 female) were enrolled in the study. The demographic characteristics are shown in Table 1. All surgical procedures and postoperative follow-ups were uneventful. Complications, including severe dry eye, haze, edema, or infection were not observed. At postoperative 6 months, the efficacy and safety index were 1.04 ± 0.15 and 1.08 ± 0.18, respectively.

**TABLE 1:** Demographic and topographic data.

Variables	Mean ± SD	Range
Age (year)	24.38 ± 4.76	18 to 35
MRSE (D)	−6.23 ± 2.06	−10.50 to −1.88
K1 (D)	42.63 ± 1.42	39.2 to 45.1
K2 (D)	44.05 ± 1.58	40.4 to 47.9
Km (D)	43.33 ± 1.46	39.8 to 46.4
Pentacam-CCT (μm)	539.38 ± 30.04	480 to 612
Ablation depth	120.98 ± 24.69	59 to 159
Attempted SE (D)	−6.55 ± 2.10	−11.00 to −2.13

MRSE, manifest refraction spherical equivalent; D, diopter; K1, flat curvature power; K2, flat curvature power; Km, mean curvature power; CCT, central corneal thickness.

**FIGURE 1:**
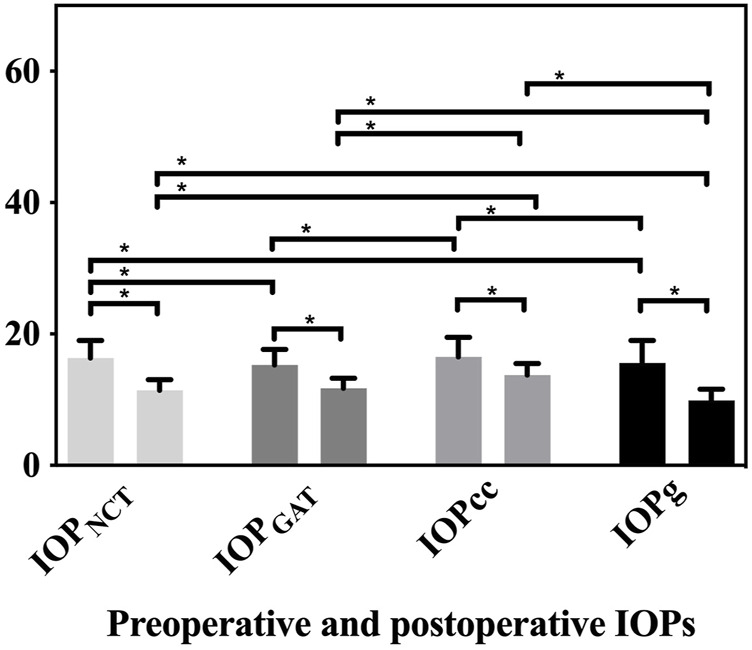
Preoperative and postoperative values of IOP_NCT_, IOP_GAT_, IOPg, and IOPcc. Significant decreases in all IOPs were detected compared to preoperative values (all *p* < 0.001). Except for IOP_NCT_ and IOPcc (*p* = 0.631) and IOP_GAT_ and IOPg (*p* = 0.382), all other preoperative IOPs differed from each other (all *p* < 0.05) (IOP_NCT_ and IOP_GAT_ *p* = 0.005, IOP_NCT_ and IOPg *p* = 0.049, IOP_GAT_ and IOPcc *p* = 0.001, and IOPcc and IOPg *p* = 0.015). After the surgery, except for IOP_NCT_ and IOP_GAT_ (*p* = 0.095), each postoperative IOP was different from other the three IOPs (all *p* < 0.001).

The IOP values are shown in Figure 1. Except for IOP_NCT_ and IOPcc (*p* = 0.631) and IOP_GAT_ and IOPg (*p* = 0.382), other preoperative IOPs were different from each other (all *p* < 0.05). Post-operation, each IOP was different from the other three IOPs (all *p* < 0.001), except for IOP_NCT_ and IOP_GAT_ (*p* = 0.095).

**FIGURE 2:**
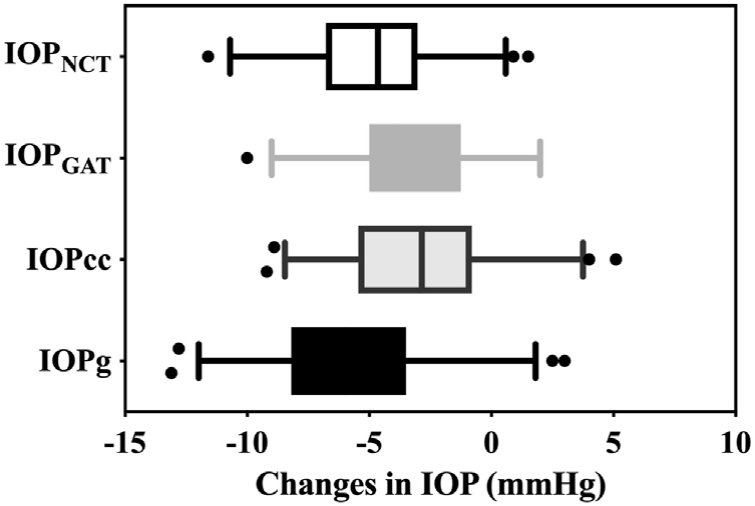
Decreases in IOP_NCT,_ IOP_GAT_, IOPg, and IOPcc. Each of the changes in IOP differed from the other three IOPs (all *p* < 0.01) (IOP_NCT_ and IOPg (*p* = 0.005), IOP_GAT_ and IOPcc (*p* = 0.006), and other *p* < 0.001)

At postoperative 6 months, significant decreases in IOP_NCT_ (4.88 ± 2.69 mmHg, 95% CI: 4.33–5.42 mmHg), IOP_GAT_ (3.58 ± 2.57 mmHg, 95% CI: 3.03–4.13 mmHg), IOPcc (2.75 ± 3.16 mmHg, 95% CI: 2.21–3.30 mmHg), and IOPg (5.72 ± 3.38 mmHg, 95% CI: 5.17–6.27 mmHg) were detected compared to preoperative values (all *p* < 0.001). All changes in IOPs differed from those of the other three IOPs (all *p* < 0.01) (Figure 2).

Information on age, preoperative and attempted SE, ablation depth, preoperative values, and postoperative changes in the central corneal thickness (CCT), K1, K2, Km, CH, and CRF values were collected in order to predict IOPs. Significant parameters in predictive models for IOPs are shown in Table 2 (all *p* < 0.05).

**TABLE 2:** Generalized linear mixed model on intraocular pressure decrease.

Measurements	Variables	Correlation coefficient	Sd	95% CI	*p* value
NCT	IOP _preoperative NCT_	0.869	0.056	0.757 to 0.981	<0.001
GAT	IOP _preoperative GAT_	0.872	0.062	0.745 to 0.999	<0.001
ORA IOPcc	IOPcc _preoperative_	0.601	0.077	0.449 to 0.753	<0.001
	CH _difference_	−1.322	0.213	−1.744 to -0.900	0.001
	CRF _difference_	1.040	0.185	0.672 to 1.408	<0.001
ORA IOPg	IOPg _preoperative_	0.998	0.057	0.876 to 1.101	<0.001
	CCT	0.019	0.006	0.006 to 0.032	0.003
	CRF _difference_	0.995	0.189	0.620 to 1.369	<0.001
	CRF _preoperative_	−1.139	0.197	−1.529 to -0.749	<0.001

CCT, central corneal thickness; CI, confidence interval; CH, corneal hysteresis; CRF, corneal resistance factor.

Predictive models are given in Table 3, and *R* ^2^ values were 67.5% (IOP_NCT_), 64.5% (IOP_GAT_), 78.7% (IOPcc), and 82.0% (IOPg).

**TABLE 3:** Predictive equations for corrected intraocular pressure after SMILE.

Measurements	Full equation	*R* ^2^
NCT	IOP _corrected NCT_ = IOP _measured NCT_+ 0.869 (IOP _preoperative NCT_) -9.284	0.675
GAT	IOP _corrected GAT_ = IOP _measured GAT_+ 0.872 (IOP _preoperative GAT_) -9.764	0.645
ORA IOPcc	IOPcc _corrected_ = IOPcc _measured_ + 0.601 (IOPcc _preoperative_) -1.322 (CH _difference_) +1.040 (CRF _difference_) -8.028	0.787
ORA IOPg	IOPg _corrected_ = IOPg _measured_ + 0.988 (IOPg _preoperative_)+ 0.995 (CRF _difference_) +0.019 (CCT _preoperative_) - 1.139 (CRF _preoperative_) -12.148	0.820

CCT, central corneal thickness; CH, corneal hysteresis; CRF, corneal resistance factor.

**FIGURE 3:**
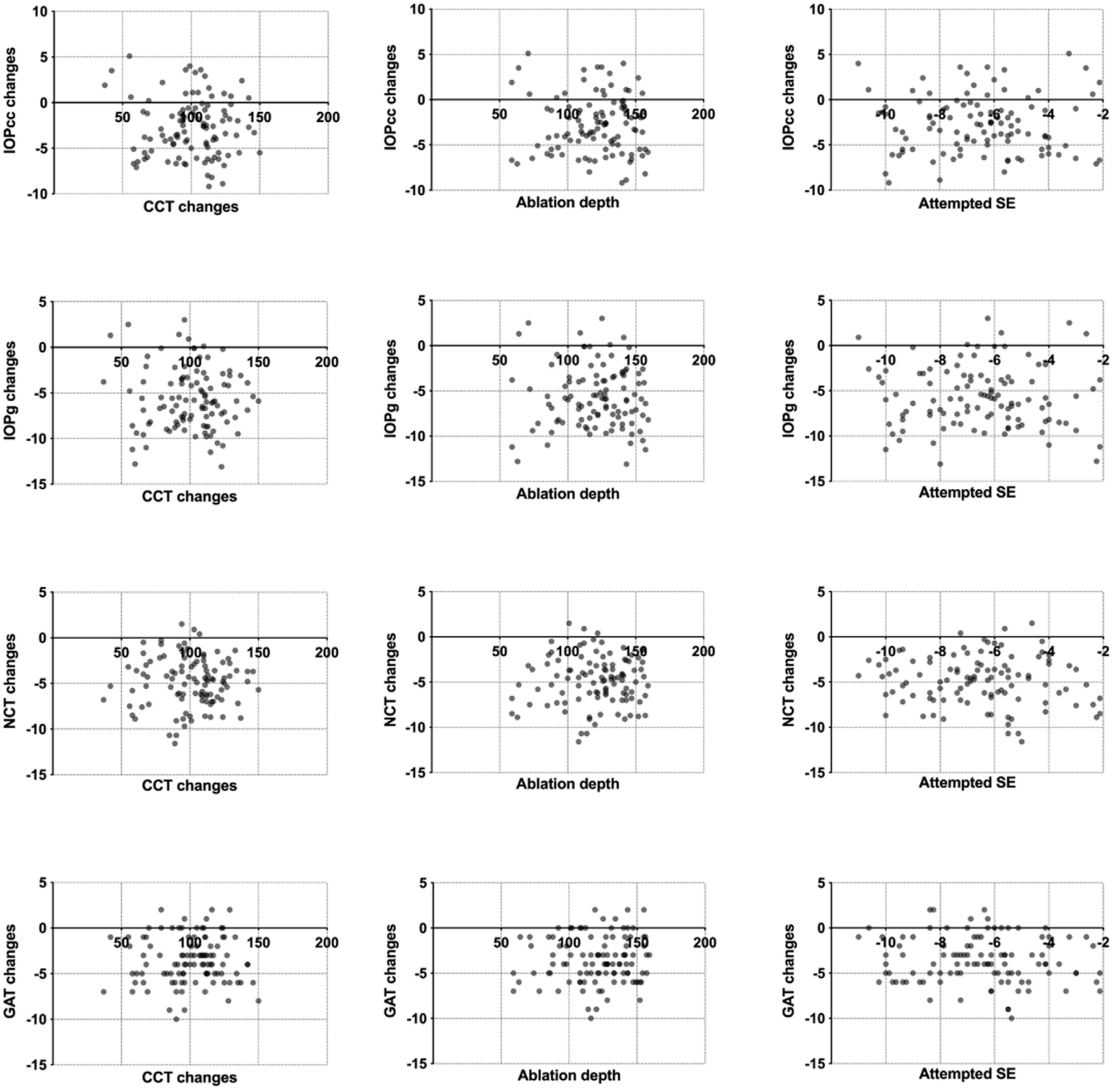
Scatterplots between IOP_NCT,_ IOP_GAT_, IOPg and IOPcc, and CCT changes, ablation depth, and attempted SE.

The preoperative IOP was the only significant parameter in the predictive models for IOP_NCT_ and IOP_GAT_ (the scatterplots between IOP changes and CCT changes, ablation depth, and attempted SE are shown in Figure 3). Thus, considering that the normal range of IOP (10–21 mmHg) is well-known, it is possible to calculate the confidence band and prediction band of the postoperative IOP_NCT_ and IOP_GAT_ (Lenhoff et al., 1999). Table 4 and Table 5 show the estimated confidence band and prediction band of postoperative IOP_NCT_ and IOP_GAT_ with preoperative IOP set as an integral.

**TABLE 4:** Estimated confidence band and prediction band of postoperative IOP_NCT_.

Preoperative IOP	95% CI of the confidence band		95% CI of the prediction band	
	Lower limit	Upper limit	Lower limit	Upper limit
10	9.8	11.4	7.4	13.7
11	10.1	11.4	7.6	13.9
12	10.3	11.4	7.7	14.0
13	10.5	11.5	7.9	14.1
14	10.7	11.5	8.0	14.2
15	10.9	11.6	8.2	14.3
16	11.1	11.7	8.3	14.4
17	11.2	11.8	8.4	14.6
18	11.3	12.0	8.6	14.7
19	11.3	12.2	8.7	14.9
20	11.4	12.4	8.8	15.0
21	11.4	12.6	8.9	15.1

CI, confidence interval.

**TABLE 5:** Estimated confidence band and prediction band of postoperative IOP_GAT_.

Preoperative IOP	95% CI of the confidence band		95% CI of the prediction band	
	Lower limit	Upper limit	Lower limit	Upper limit
10	10	12	8	14
11	11	12	8	14
12	11	12	8	14
13	11	12	8	15
14	11	12	8	15
15	11	12	9	15
16	11	12	9	15
17	12	12	9	15
18	12	13	9	15
19	12	13	9	15
20	12	13	9	15
21	12	13	9	16

CI, confidence interval.

## Discussion

Changes in corneal structures influence the evaluation of intraocular pressure, and both changes in the corneal structure and IOP evaluation differ after various corneal refractive surgeries (Chang and Stulting, 2005; Kohlhaas et al., 2006; Yang et al., 2006; Pepose et al., 2007; Johannesson et al., 2012; Han et al., 2013; Shen et al., 2014; Schallhorn et al., 2015; Sales-Sanz et al., 2016). Because a lenticule is extracted through a 2-mm incision during a SMILE procedure, the integrity of the corneal tissue (including Bowman’s layer) and corneal biomechanics are highly preserved. Thus, predictive models for IOP evaluation after a SMILE procedure is worthy of discussion.

In this study, significant decreases exist in all four IOP measurements compared to preoperative values. Previous studies on other refractive surgeries also found that IOP values decreased (Chang and Stulting, 2005; Kohlhaas et al., 2006; Yang et al., 2006; Pepose et al., 2007; Johannesson et al., 2012; Han et al., 2013; Schallhorn et al., 2015; Sales-Sanz et al., 2016). IOP value changes in this study are similar to those in previous studies on SMILE (Li et al., 2016; Shen et al., 2016; Hosny et al., 2017; Chen et al., 2018; Chen et al., 2020). These results suggest an underestimation of IOP measurement after refractive surgeries.

Ablation depth commonly plays an important role in predictive models after refractive surgeries, like LASIK and PRK. It is interesting to note that ablation depth fails to contribute to the predictive models in this study. Our results were similar to those of other studies on changes in IOP after SMILE, in that ablation depth, preoperative SE, attempted SE, CCT changes, differences between preoperative and postoperative K1, K2, and Km parameters were not parameters in all predictive models (Li et al., 2016; Shen et al., 2016; Hosny et al., 2017). The unusual phenomenon might be attributed to distinctive corneal structure changes after SMILE procedures. The integrity of the corneal tissue and the small incision leads to these changes in IOP after SMILE compared to changes after other refractive surgeries.

A similar or higher *R* ^2^ value for a predictive model of IOP_NCT_ was reported in other studies. In a study on IOP changes after LASIK in 133,752 myopic eyes, the *R* ^2^ value is 45% (Schallhorn et al., 2015). A much higher *R* ^2^ value (91%) was achieved in Yang et al. (2006)’s predictive model for IOP_NCT_ after LASIK. In our opinion, there is room for improvement in the predictive models. Although only preoperative IOP_NCT_ and IOP_GAT_ played a significant role in the predictive models, it seems that there should be more parameters involved, such as corneal biomechanics (Xin et al., 2022).

However, since only one variable predicts postoperative IOP_NCT_ and IOP_GAT_, it is convenient to apply the predictive models in clinical practice by calculating the estimated confidence band and prediction band. The confidence band and prediction band can be easily understood as the mean and evaluation range of a postoperative IOP value when the preoperative IOP is a known point. For example, if the preoperative IOP_NCT_ of an eye that underwent a SMILE procedure was 15 mmHg, then the 95% CI of the mean postoperative IOP_NCT_ would be 10.9–11.6 mmHg, and the 95% CI of the postoperative IOP_NCT_ range would be 8.2–14.3 mmHg. In addition, the application of all predictive models was limited to patients of SMILE procedures with normal IOPs. Thus, for these eyes, as shown in Table 3, Table 4, and Table 5, the 95% CI of the postoperative IOP_NCT_ and IOP_GAT_ range was 7.4–15.1 mmHg and 8–16 mmHg, respectively. In practice, if the postoperative IOP_NCT_ of an eye that underwent a SMILE procedure was 18 mmHg, hypertension would be highly suspected.

Although there was no difference between IOP_GAT_ and IOPg at pre-operation, the decrease in IOP_GAT_ was smaller than in IOPg after SMILE. This suggests that IOP_GAT_ is more stable than IOPg, which is consistent with a study on IOP changes after LASIK (Pepose et al., 2007). The decrease in IOPcc was the smallest among all four IOPs, supporting previous observations that IOPcc is less affected by changes in the corneal structure (Medeiros and Weinreb, 2006; Li et al., 2016). In this study, predictive models explained 67.5% of IOP_NCT_ variance, 64.5% of IOP_GAT_ variance, 78.7% of IOPcc variance, and 82.0% of IOPg variance. *R* ^2^ values of IOPcc (78.7%) and IOPg (82.0%) were much higher than those of IOP_NCT_ (67.5%) and IOP_GAT_ (64.5%). We speculate that this might relate to the corneal biomechanics, which were present in both predictive models of IOPg and IOPcc.

To obtain the most accurate IOP measurements, all measurements were performed between 9 and 12 o’clock in the morning, avoiding IOP fluctuation (Kohlhaas et al., 2006). Moreover, considering that the flap depth affects IOP measurements, all cap depths were set to 120 μm (Lin et al., 2016; Liu et al., 2018). In addition, effective refractive outcomes are the basis for developing predictive models. In this study, the efficacy and safety index were 1.04 ± 0.15 and 1.08 ± 0.18, respectively. The outcomes were consistent with previous studies (Li et al., 2016).

Limited sample size is a potential limitation to this study. A larger sample size would be more convincing.

In conclusion, predictive models for IOP measurements after SMILE procedures can be helpful in clinical practice. It is worthwhile to enhance these models in further studies.

## Data Availability

The raw data supporting the conclusion of this article will be made available by the authors, without undue reservation.
